# Psychometric assessment of scales for a Model of Goal Directed Vegetable Parenting Practices (MGDVPP)

**DOI:** 10.1186/1479-5868-10-110

**Published:** 2013-09-22

**Authors:** Tom Baranowski, Alicia Beltran, Tzu-An Chen, Debbe Thompson, Teresia O’Connor, Sheryl Hughes, Cassandra Diep, Janice Baranowski

**Affiliations:** 1 USDA/ARS Children’s Nutrition Research Center, Baylor College of Medicine, Department of Pediatrics, 1100 Bates Street, Houston 77030-2600 TX, USA

**Keywords:** Vegetable, Parenting practices, Psychometrics, Model of goal directed behavior, Self determination theory

## Abstract

**Background:**

Vegetable intake has been related to lower risk of chronic illnesses in the adult years. The habit of vegetable intake should be established early in life, but many parents of preschoolers report not being able to get their child to eat vegetables. The Model of Goal Directed Behavior (MGDB) has been employed to understand vegetable parenting practices (VPP) to encourage a preschool child’s vegetable intake. The Model of Goal Directed Vegetable Parenting Practices (MGDVPP) provides possible determinants and may help explain why parents use effective or ineffective VPP. Scales to measure effective and ineffective vegetable parenting practices have previously been validated. This manuscript presents the psychometric characteristics and factor structures of new scales to measure the constructs in MGDVPP.

**Methods:**

Participants were 307 parents of preschool (i.e. 3 to 5 year old) children, used for both exploratory (EFA) and confirmatory factor analyses (CFA). Data were collected via an internet survey. First, EFA were conducted using the scree plot criterion for factor extraction. Next, CFA assessed the fit of the exploratory derived factors. Then, classical test theory procedures were employed with all scales. Finally, Pearson correlations were calculated between each scale and composite effective and ineffective VPP as a test of scale predictive validity.

**Results:**

Twenty-nine subscales (164 items) within 11 scales were extracted. The number of items per subscale ranged from 2 to 13, with three subscales having 10 or more items and 12 subscales having 4 items or less. Cronbach’s alphas varied from 0.13 to 0.92, with 17 being 0.70 or higher. Most alphas <0.70 had only three or four items. Twenty-five of the 29 subscales significantly bivariately correlated with the composite effective or ineffective VPP scales.

**Discussion:**

This was the initial examination of the factor structure and psychometric assessment of MGDVPP scales. Most of the scales displayed acceptable to desirable psychometric characteristics. Research is warranted to add items to those subscales with small numbers of items, test their validity and reliability, and characterize the model’s influence on child vegetable consumption.

## Background

High vegetable intake has been inversely related to risk of heart disease and stroke, likely with several cancers [1], and obesity in the adult years [2]. Vegetable intake tracks from the earliest years [3], supporting the likelihood that preference for [4] and habit of vegetable intake is established early in life, even as early as the preschool years [5].

Parents are believed to be important influences on child dietary intake, especially in the preschool years [6]. However, many parents of preschoolers report difficulties in getting their child to eat vegetables [7]. Separate vegetable parenting practices (VPP) dimensions have recently been identified that are likely effective (E) VPP for getting a child to eat and enjoy vegetables (e.g. Effective Responsiveness “I tell my child that vegetables taste good”) and ineffective (I) VPP in getting a child to eat vegetables (e.g. Ineffective Responsiveness “I give my child something to eat or drink if they are bored”) [8]. Many parents of preschoolers use both EVPP and IVPP, suggesting that they are not aware of practices that are likely to be effective or not [8].

To design effective intervention programs we need to understand why parents might employ EVPP and IVPP. The existing research predicting specific feeding parenting practices has focused on psycho-pathological or sociological factors. For example, stress and depression predicted impaired feeding specific parenting, while perceived social support predicted improved parenting [9]. Higher levels of maternal education were associated with mother’s higher use of controlling and lower use of emotional feeding practices [10]. Mother’s parenting satisfaction was associated with less pressure on the child to eat and less food restriction [11]. The next step in this line of investigation is to more narrowly focus the behavior (e.g. parenting practices to enhance child vegetable intake) and incorporate a model to identify the likely psychosocial predictors of the behavior.

**Figure 1:**
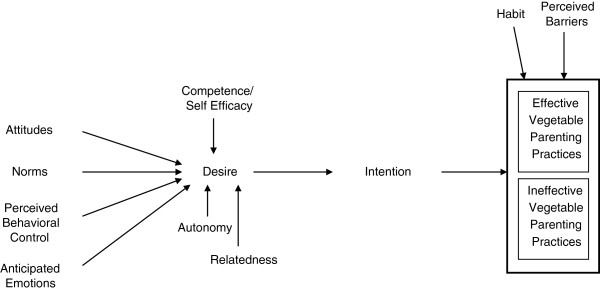
A model of goal directed vegetable parenting practices.

A Model of Goal Directed Behavior (MGDB) obtained high levels of adult health behavior predictiveness [12-14] by incorporating “anticipated emotions” into the Theory of Planned Behavior (TPB), and inserting “desire” between the psychosocial predictors and intentions [12,15]. Since “desire” was operationalized to embody “intrinsic motivation” [12,15], constructs from Self Determination Theory that contribute to intrinsic motivation (autonomy, competence, relatedness) [16] were added to the model. Competence is similar to Social Cognitive Theory’s Self Efficacy construct [17-19]. Since habit (i.e. automated behavior) [20] and barriers [21] were strongly related to behavior, incorporating these variables should enhance predictiveness and understanding (See Figure 1). This previously unpublished enhanced MGDB provided the conceptual framework for this study.

Qualitative research conducted by the authors was used to generate items to populate scales within this model [22]. The present manuscript reports preliminary psychometric analyses of newly generated items for a Model of Goal Directed Vegetable Parenting Practices (MGDVPP) scales and subscales. To our knowledge, this is the first report of the psychometrics of scales for MGDVPP.

## Methods

### Overview

Intensive qualitative interviews were conducted with parents of preschool children to generate items for MGDVPP scales [22]. An internet survey including 192 items covering 11 scales was then employed using Survey Monkey [23]. Exploratory factor analyses were conducted using the scree plot criterion for factor extraction. Next, confirmatory factor analyses were conducted to test the fit of the exploratory derived factors. Then, classical test theory procedures (i.e. item means, standard deviations, corrected item-total correlations, average inter-item correlations, Cronbach’s alpha) were employed with all empirically determined subscales. Last, bivariate Pearson correlations were calculated between each subscale and composite EVPP and IVPP as a test of predictive validity.

### Sample recruitment

An internet survey was announced in a Children’s Nutrition Research Center (CNRC) newsletter distributed to 25,000 recipients; fliers were posted on participant volunteer billboards around the Texas Medical Center, public libraries and YMCA’s. We also sent personal emails to the CNRC list of volunteers, and listed the study on the Baylor College of Medicine (BCM) volunteer website. Inclusionary criteria were being a parent of a preschool child, able to read and write English, and having the child spend most of the time with that caregiver. Access to the internet survey implied access to both a computer and an internet connection. Given the low risk nature of the study, selecting the “participate” button in the survey was taken as evidence of consent. The Institutional Review Board of the Baylor College of Medicine reviewed and approved the research protocol. This sample was used for both the Exploratory and Confirmatory Factor Analyses.

### Item generation

Qualitative telephone interviews were conducted using a semi-structured script with a multicultural sample of parents of 3–5 year old children [22]. The interview script consisted of twelve open-ended questions and several structured follow up questions, prompts, and probes. Interviews were taped; and verbatim transcripts created, coded and analyzed using thematic analysis. MGDB [12,15] provided the theoretical framework and guided the questionnaire development and interpretation of results. Themes were identified from the transcripts and transformed into items for a questionnaire. Cognitive interviews were conducted to assess parent understanding of item wording; as a result, some items were simplified and others deleted. Based on theory, the 192 items were divided across 11 scales. Three category responses were employed for all scales given our repeated finding using item response modeling that respondents generally effectively used only two or three response categories [17-19].

Eighteen attitude items were generated, each starting with the stem: “If my child started eating more vegetables on most days…” A three category response was employed (1 = Disagree; 2 = Neither Agree nor Disagree; 3 = Agree). (See individual items in Table 1.)

**Table 1:** Items and factor loadings from an exploratory three factor solution of attitudes toward use of vegetable parenting practices and confirmatory factory analysis model fit criteria

**Attitude items**	**Factor 1 loadings**	**Factor 2 loadings**	**Factor 3 loadings**
	**Health benefits of vegetables**	**Negative effects of vegetables**	**Benefits of vegetables other than health**
***Health benefits of vegetables:****“If my child started eating more vegetables on most days, my child would …”*			
…have better teeth.	**.757**	-.046	-.029
…think better.	**.742**	-.012	.032
…live longer.	**.660**	-.129	.075
…have more energy to play.	**.590**	.131	.147
…have fewer stomach problems, like constipation and stomach aches.	**.543**	.055	.165
…be healthier.	**.448**	-.066	.325
***Negative effects of vegetables:****“If my child started eating more vegetables on most days, my child would …”*			
…be exposed to germs on vegetables.	.042	**.696**	-.003
…have more stomach problems, like diarrhea or gas.	-.061	**.661**	-.012
…be exposed to unhealthy chemicals on vegetables.	.024	**.614**	-.055
…be too thin.	-.074	**.582**	-.095
…make me spend too much on groceries.	.063	**.537**	-.189
…gain too much weight.	-.044	**.494**	.029
***Benefits of vegetables other than health:****“If my child started eating more vegetables on most days, my child would …”*			
…be exposed to a variety of foods.	.006	-.023	**.765**
…be exposed to new foods.	.100	-.024	**.721**
…learn better eating habits.	.241	-.078	**.685**
…get more vitamins.	.235	-.112	**.581**
Eigenvalue	3.487	2.403	1.509
Variance explained	19.4%	13.4%	8.4%
	Pearson correlation		
***Health benefits of vegetables***		−0.020	0.317***
***Negative effects of vegetables***			−0.140*
***Benefits of vegetables other than health***			
*Model fit indices from a three factor confirmatory factor analysis*			
*X*^*2*^	145.517		
df	101		
*p*	0.003		
RMSEA	0.038		
SRMR	0.099		
CFI	0.962		
TLI	0.955		
***Items not included in a final solution:****“If my child started eating more vegetables on most days, my child would …”*			
…try to get me to eat more vegetables.	.324	.374	.202
…set a good example for others.	.381	.009	.261

**Legend**: Response Scale: 1 = Disagree, 2 = Neither Agree nor Disagree, 3 = Agree; for the Pearson correlations between subscales: * = p < 0.05, *** = p < 0.001.

Items were created for two different types of norms. Descriptive norms identified the respondents’ perceptions of what parents and children were currently doing in regard to the child’s eating of vegetables. We asked the respondents’ perception of the extent to which most parents get their child to eat more vegetables, to have their child eat enough vegetables, and the extent to which most children eat vegetables. Parents were asked to select from a three category response option which included: 1 = Disagree; 2 = Neither Agree nor Disagree; 3 = Agree, for each statement. Closer to the original formulation for TPB, normative expectations identified what the respondent believed other people expected them to do, and the extent to which the respondent wanted to please those people. Given the complexities of modern family structures and living arrangements, different respondents are likely responsive to the expectations of people in different social roles. To reduce this complexity we asked the respondent to identify “the three most important people who influence your decisions about your child in a good, or a bad way” from a menu (see Table 2). For each of these three role players, the respondent was asked to respond to two questions: “It is important to my [role person] that my child eats more vegetables”; and “It is important to me to please my [role person] when it comes to getting my child to eat more vegetables”. Parents were asked to select from a three category response (1 = Disagree; 2 = Neither Agree nor Disagree; 3 = Agree) for each statement. (See individual items in Table 3.)

**Table 2:** Frequency and percents of the first, second, and third most important person “…who influences your decisions about your child in a good, or a bad, way”

	**Most important**		**Second most important**		**Third most important&**	
	**n**	**%**	**n**	**%**	**n**	**%**
Spouse or partner	155	50.5	63	20.5	12	4.7
Mother	85	27.7	66	21.5	25	9.9
Mother-in-law	1	.3	10	3.3	20	7.9
Father	17	5.5	28	9.1	18	7.1
Father-in-law	-	0.0	1	.3	1	.4
Caregiver/Babysitter/Nanny	4	1.3	12	3.9	9	3.6
Grandmother	15	4.9	13	4.2	16	6.3
Grandfather	4	1.3	5	1.6	4	1.6
Sister/Brother	8	2.6	15	4.9	19	7.5
Sister-in-law/Brother-in-law	-	0.0	1	.3	6	2.4
Close friend	10	3.3	24	7.8	17	6.7
Teacher	8	2.6	15	4.9	13	5.1
No other person	-	0.0	54	17.6	93	36.8

&Due to concerns for the complications from missing data, we included responses in regard to the second most important person, but not the third.

**Table 3:** Items and factor loadings from an exploratory two factor solution of norms toward use of vegetable parenting practices and confirmatory factor analysis model fit criteria

**Norm items**	**Factor 1 loadings**	**Factor 2 loadings**
	**Descriptive norms**	**Normative expectations**
***Descriptive norms***		
Most parents have their child eat enough vegetables&	**.865**	.140
Most children eat enough vegetables	**.840**	.058
Most parents try to get their child to eat more vegetables	**.406**	.033
***Normative expectations***&&^,^ &&&		
It is important to the [Most Important Person] that my child eats more vegetables. x It is important to me to please the [Most Important Person] when it comes to getting my child to eat more vegetables.	.052	**.885**
It is important to the [Second Most Important Person] that my child eats more vegetables. x It is important to me to please the [Second Most Important Person] when it comes to getting my child to eat more vegetables.	.128	**.875**
Eigenvalue	1.945	1.259
Variance explained	38.9%	25.2%
	Pearson correlation	
***Descriptive norms***		0.087
***Normative expectations***		
*Model Fit Indices from a Two Factor Confirmatory Factor Analysis*		
*X*^*2*^	0.728	
df	1	
*p*	0.394	
RMSEA	0.000	
SRMR	0.018	
CFI	1.000	
TLI	1.007	

&This item was included in the exploratory factor analysis, but was excluded from the confirmatory factor analysis to enable the analysis to converge.

&&Due to concerns for the complications from missing data, we included responses in regard to the second most important person, but not the third. See Table 2.

&&&Exploratory factor analyses of the norms items were conducted in two ways. First, we included the three descriptive norm statements and two normative expectations statements about the important person expecting the child to eat vegetables (data not shown). Second, we included the three descriptive norm statements and the two normative expectation statements, but the values for the latter two were multiplied by the extent to which the respondent wanted to please the important person (possible range of scores: 1 to 9). The factor structure with the importance items multiplied by the extent of desire to please yielded the most interpretable structure (Table 3).

Thirty perceived behavioral control items were generated starting with the stem “How easy would it be to get my child to eat more vegetables if I…”, using a three category difficulty response (1 = Difficult; 2 = Neither Easy nor Difficult; 3 = Easy). (See individual items in Table 4.)

**Table 4:** Items and factor loadings from an exploratory three factor solution of perceived behavioral control toward use of vegetable parenting practices and confirmatory factor analysis model fit criteria

**Perceived behavioral control items**	**Factor 1 loadings**	**Factor 2 loadings**	**Factor 3 loadings**
	**Control over positive influences on vegetable consumption**	**Control over negative influences on vegetable consumption**	**Control over negative parenting practices**
***Perceived behavioral control of positive influences on vegetable consumption:****“How easy would it be to get my child to eat more vegetables if I…”*			
…ask them to select vegetables at the grocery store.	**.781**	-.037	-.055
…show them I enjoy eating vegetables.	**.698**	.000	.159
…ask them to help with vegetable preparation.	**.659**	-.117	-.019
…tell them eating vegetables will make them strong and healthy.	**.642**	.020	.131
…tell them that vegetables taste good.	**.627**	.060	-.034
…praise them when I see them eat vegetables.	**.614**	.001	.006
…ask them to choose their vegetables for meals and snacks.	**.614**	-.034	-.036
…schedule meals for them.	**.609**	-.056	.253
…mix vegetables with their favorite foods.	**.572**	-.048	.074
…encourage them to try a couple of bites of a vegetable.	**.525**	-.111	.078
…allow them to serve themselves vegetables.	**.522**	.023	-.071
…tell them that their favorite cartoon characters eat vegetables.	**.501**	.106	.097
…limit cookies, chips and candy in our house.	**.419**	-.138	.224
***Perceived behavioral control of negative influences on vegetable consumption:****“How easy would it be to get my child to eat more vegetables if I…”*			
…give them something sweet to eat or drink if they are upset.	-.096	**.732**	.119
…keep lots of sweets (candy, ice cream, cake, pies, pastries) in our house.	-.072	**.727**	.025
…give them something sweet to eat or drink if they are bored.	-.084	**.693**	.165
…allow them to drink sweet drinks.	-.045	**.662**	-.116
…drink soda in front of them.	.045	**.603**	-.073
…let them eat between meals whenever they want.	-.108	**.582**	.166
…give them multiple servings of food regardless of whether they have eaten their vegetable.	.132	**.573**	.047
…take multiple helpings of other food in front of them.	.186	**.537**	.092
…am so busy that I don’t notice when they talk about the food.	.008	**.504**	.157
…do not respond when they ask about the food.	-.103	**.505**	.350
…let them watch TV at meals.	-.165	**.424**	.106
***Perceived behavioral control of negative parenting practices:****“How easy would it be to get my child to eat more vegetables if I…”*			
…insist they sit at the table until they eat their vegetables.	.137	.140	**.615**
…beg them to eat vegetables.	-.061	.230	**.573**
…make them feel guilty when they don’t eat vegetables.	-.076	.347	**.546**
…promise them something other than food if they finish their vegetables.	.130	-.001	**.519**
Eigenvalue	5.293	4.613	1.466
Variance explained	17.6%	15.4%	4.9%
	Pearson Correlation		
***Perceived behavioral control of positive influences on vegetable consumption***		−0.071	0.143*
***Perceived behavioral control of negative influences on vegetable consumption***			0.373***
***Perceived behavioral control of negative parenting practices***			
*Model fit indices from a three factor confirmatory factor analysis*			
*X*^*2*^	494.203		
df	342		
*p*	<0.001		
RMSEA	0.038		
SRMR	0.085		
CFI	0.956		
TLI	0.951		
***Items not included in a final solution:****“How easy would it be to get my child to eat more vegetables if I…”*			
…cut back on how often we eat at restaurants or fast food places.	.314	-.123	.317
…tell them they will get a stomach-ache if they eat too many cookies, chips and candies instead of vegetables.	.274	.177	.377

**Legend**: Response Scale: 1 = Disagree, 2 = Neither Agree nor Disagree, 3 = Agree; for the Pearson correlations between subscales: * = p < 0.05, *** = p < 0.001.

Anticipated Emotion items systematically varied types of vegetables served (i.e. usual, new, liked, disliked) with eating behavior (ate it, refused it), since we believed consistent and inconsistent service and behavior would lead to diverse meaningful emotional responses. Thirty-two anticipated emotion items were generated starting with four different stems: “If I served my child a new vegetable and they ate it, I would feel…”; “If I served my child a new vegetable and they refused to eat it, I would feel…”; “If I served my child a vegetable that they liked, and they refused to eat it, I would feel…”; “If I served my child a vegetable that I knew they disliked, and they ate it, I would feel…”. Three agreement response categories were offered (1 = Disagree; 2 = Neither Agree nor Disagree; 3 = Agree). (See individual items in Table 5.)

**Table 5:** Items and factor loadings from an exploratory four factor solution of anticipated emotions toward use of vegetable parenting practices and confirmatory factor analysis model fit criteria

**Anticipated emotions items**	**Factor 1 loadings**	**Factor 2 loadings**	**Factor 3 loadings**	**Factor 4 loadings**
	**Negative child behavior with positive parent emotional response**	**Positive child behavior with negative parent emotional response**	**Negative child behavior with negative parent emotional response**	**Positive child behavior with positive parent emotional response**
***Positive parent emotional response to child vegetable refusal :****“If I served my child a new vegetable and they refused to eat it, I would feel…”*				
…happy.	**.800**	.109	-.147	.010
…excited.	**.776**	.104	-.093	.096
…proud.	**.721**	.103	-.176	.005
…upset.	**.559**	.172	.286	-.322
*“If I served my child a new vegetable that they liked, and they refused to eat it, I would feel…”*				
…excited.	**.774**	.312	-.136	.066
…happy.	**.770**	.313	-.192	.047
…proud.	**.763**	.290	-.175	.060
…pleased.	**.735**	.314	-.161	.036
***Negative parent emotional response to child vegetable acceptance:****“If I served my child a vegetable that I knew they disliked, and they ate it, I would feel…”*				
…upset.	.284	**.806**	.079	-.083
…frustrated.	.350	**.739**	.063	.028
…disappointed.	.283	**.730**	.086	-.023
…concerned.	.150	**.702**	.153	.030
***Negative parent emotional response to child vegetable refusal:****“If I served my child a new vegetable and they refused to eat it, I would feel…”*				
…frustrated.	-.096	.011	**.707**	.028
…upset.	.031	.143	**.655**	.076
…concerned.	.047	.133	**.513**	.228
…disappointed.	-.254	.007	**.513**	.164
*“If I served my child a vegetable that they liked, and they refused to eat it, I would feel…”*				
…upset.	.024	.124	**.681**	.065
…frustrated.	-.210	-.037	**.630**	-.054
…disappointed.	-.196	-.027	**.566**	.168
…concerned.	.010	-.073	**.433**	.116
***Positive parent emotional response to child vegetable acceptance:****“If I served my child a new vegetable and they ate it, I would feel…”*				
…happy.	-.038	.029	.137	**.692**
…excited.	-.056	.077	.169	**.689**
…proud.	-.052	.067	.100	**.551**
*“If I served my child a vegetable that I knew they disliked, and they ate it, I would feel…proud.”*	.089	-.350	.259	**.594**
Eigenvalue	7.755	4.004	3.096	1.880
Variance explained	24.2%	12.5%	9.7%	5.9%
	Pearson correlation			
***Positive parent emotional response to child vegetable refusal***		0.444***	−0.178**	−0.094
***Negative parent emotional response to child vegetable acceptance***			0.069	−0.100
***Negative parent emotional response to child vegetable refusal***				0.271***
***Positive parent emotional response to child vegetable acceptance***				
*Model fit indices from a four factor confirmatory factor analysis*				
*X*^*2*^	699.692			
df	235			
*p*	<0.001			
RMSEA	0.08			
SRMR	0.113			
CFI	0.988			
TLI	0.986			
***Items not included in a final solution***				
*“If I served my child a new vegetable and they ate it, I would feel…”*				
…pleased.	-.468	.110	-.107	.246
…disappointed.	.516	.027	.185	-.431
…frustrated.	.510	.152	.261	-.425
…concerned.	.288	.371	.150	-.044
*“If I served my child a new vegetable and they refused to eat it, I would feel…pleased.”*	.729	.032	-.108	-.011
*“If I served my child a vegetable that I knew they disliked, and they ate it, I would feel…”*				
…happy.	.103	-.438	.253	.576
…excited.	.136	-.436	.319	.505
…pleased.	.017	-.558	.150	.527

**Legend**: Response Scale: 1 = Disagree, 2 = Neither Agree nor Disagree, 3 = Agree; for the Pearson correlations between subscales: ** = p < 0.01, *** = p < 0.001

Twenty habit items were generated starting with the stem “Without thinking about it…”, using a three category frequency response (1 = Always, 2 = Sometimes, 3 = Never). (See individual items in Table 6.)

**Table 6:** Items and factor loadings from an exploratory four factor solution of habit toward use of vegetable parenting practices and confirmatory factor analysis model fit criteria

**Habit Items**	**Factor 1 loadings**	**Factor 2 loadings**	**Factor 3 loadings**	**Factor 4 loadings**
	**Active child involvement in vegetable selection**	**Controlling vegetable practices**	**Positive vegetable environment**	**Positive vegetable communications**
***Habit of active child involvement in vegetable selection:****“Without thinking about it I…”*				
…ask my child to help select vegetables at the grocery store.	**.818**	.048	.120	.016
…ask my child to help with vegetable preparation.	**.782**	.098	.055	.043
…ask my child to choose the vegetables for meals and snacks.	**.776**	.012	.141	.269
…allow my child to serve themselves vegetables.	**.732**	.073	-.005	.004
…serve several vegetables and let my child decide which one they would eat.	**.644**	-.045	.143	.097
…place vegetables where my child can easily reach them.	**.513**	.011	.312	.115
***Habit of controlling vegetable practices:****“Without thinking about it I…”*				
…yell at my child for not eating their vegetables.	-.047	**.729**	-.169	-.071
…keep my child from going to play if they don’t eat their vegetables.	.058	**.716**	.053	.055
…reward my child with sweets if they eat their vegetables.	-.002	**.692**	-.010	.031
…tell my child how much effort it took to make the vegetable dish.	.185	**.581**	.033	.113
…keep my child from having sweets if they don’t finish their vegetables.	.003	**.535**	.319	.180
***Habit of positive vegetable environment:****“Without thinking about it I…”*				
…include vegetables with most meals.	.140	.024	**.786**	.187
…show my child that I enjoy eating vegetables.	.134	.045	**.731**	.143
…serve meals for my family to eat together.	.177	.095	**.613**	.094
***Habit of positive vegetable communications:****“Without thinking about it I…”*				
…praise my child when I see them eat vegetables.	.023	.079	-.014	**.702**
…tell my child eating vegetables will make them strong and healthy.	.091	.234	.169	**.657**
…tell my child that vegetables taste good.	.168	-.110	.228	**.653**
…encourage my child to try a couple of bites of a vegetable.	.038	-.024	.377	**.550**
…tell my child that their favorite cartoon characters eat vegetables.	.365	.257	-.152	**.465**
Eigenvalue	4.536	2.315	1.963	1.214
Variance explained	22.7%	11.6%	9.8%	6.1%
	Pearson correlation			
***Habit of active child involvement in v selection***		0.143*	0.341***	0.370***
***Habit of controlling v practices***			0.121*	0.275***
***Habit of positive v environment***				0.354***
***Habit of positive v communications***				
*Model fit indices from a four factor confirmatory factor analysis*				
*X*^*2*^	264.267			
df	142			
*p*	<0.001			
RMSEA	0.053			
SRMR	0.088			
CFI	0.956			
TLI	0.947			
***Item not included in a final solution***				
Without thinking about it I…allow my child to drink sweet drinks.	-.020	.266	-.333	.034

**Legend**: Response Scale: 1 = Disagree, 2 = Neither Agree nor Disagree, 3 = Agree; for the Pearson correlations between subscales: * = p < 0.05, *** = p < 0.001.

Twenty-one competence/self efficacy items were generated with a three category response (1 = Not Sure, 2 = Somewhat Sure, 3 = Sure). (See individual items in Table 7.)

**Table 7:** Items and factor loadings from an exploratory two factor solution of competence/self efficacy toward use of vegetable parenting practices and confirmatory factor analysis model fit criteria

**Competence/self efficacy items**	**Factor 1 loadings**	**Factor 2 loadings**
	**Strong self efficacy**	**Weak self efficacy**
***Advanced vegetable parenting self efficacy***		
I can get my child to eat vegetables at most dinners.	**.761**	.109
I can get my child to eat vegetables at most lunches.	**.742**	.048
I can get my child to eat vegetables at most snacks.	**.726**	.034
I can serve 3 portions of vegetables most days of the week, even when I am stressed.	**.666**	.208
I can serve 3 portions of vegetables most days of the week.	**.624**	.206
I can serve 3 portions of vegetables most days a week, even when I am busy.	**.620**	.252
I can prepare vegetables in a way my child will eat them.	**.613**	.350
I can overcome problems in getting my child to eat vegetables.	**.603**	.028
***Preliminary vegetable parenting self efficacy***		
I can always have vegetables available at home so my child can eat them.	.200	**.673**
I can buy vegetables.	-.134	**.640**
I can afford vegetables.	-.064	**.622**
I can learn to prepare vegetables in different ways.	.178	**.620**
I can serve 1 portion of vegetable at dinner most days of the week.	.144	**.607**
I can buy vegetables in season.	.065	**.530**
I can find time to prepare vegetables for my child.	.364	**.517**
I can offer at least two different vegetables to my child so he can pick one.	.308	**.509**
I can cut 1 portion of vegetable and serve it with a low calorie dip for a snack at least once a week.	.238	**.411**
I can eat vegetables in front of my child even though I don’t like them.	.238	**.407**
Eigenvalue	6.156	2.158
Variance explained	29.3%	10.3%
	Pearson correlation	
***Advanced v parenting self efficacy***		0.477***
***Preliminary v parenting self efficacy***		
*Model fit indices from a two factor confirmatory factor analysis*		
*X*^*2*^	221.443	
df	129	
*p*	<0.001	
RMSEA	0.048	
SRMR	0.095	
CFI	0.982	
TLI	0.979	
***Items not included in a final solution***		
I can make vegetables that my family will eat.	.431	.483
I can buy vegetables (not French fries) for my child at a restaurant or fast food place.	.256	.332
I can cut 1 portion of vegetable and serve it with a low calorie dip for a snack, most days of the week.	.340	.314

**Legend**: Response Scale: 1 = Disagree, 2 = Neither Agree nor Disagree, 3 = Agree; for the Pearson correlations between subscales: *** = p < 0.001.

Twelve relatedness items were generated starting with the stem “If my child ate at least 3 portions of vegetables most days I would feel…”, using a three category agreement response (1 = Disagree, 2 = Neither Agree nor Disagree, 3 = Agree). (See individual items in Table 8.)

**Table 8:** Items and factor loadings from an exploratory two factor solution of relatedness toward use of vegetable parenting practices and confirmatory factor analysis model fit criteria

**Relatedness items**	**Factor 1 loadings**	**Factor 2 loadings**
	**Parent values**	**Child wellness**
***Relatedness through parent values:****“If my child ate at least 3 portions of vegetables most days I would feel…”*		
…I am respected by others.	**.811**	.180
…I am pleasing others.	**.759**	.091
…I am following my spiritual beliefs.	**.757**	.136
…closer to my child.	**.678**	.154
***Relatedness through child wellness:****“If my child ate at least 3 portions of vegetables most days I would feel…”*		
…I am a responsible parent.	.110	**.723**
…I have a healthy child.	-.002	**.696**
…I have a wholesome child.	.358	**.628**
Eigenvalue	5.728	1.165
Variance explained	47.7%	9.7%
	Pearson correlation	
***Relatedness through parent values***		0.466***
***Relatedness through child wellness***		
*Model fit indices from a two factor confirmatory factor analysis*		
*X*^*2*^	27.644	
df	13	
*p*	0.010	
RMSEA	0.061	
SRMR	0.044	
CFI	0.992	
TLI	0.987	
***Items not included in a final solution:****“If my child ate at least 3 portions of vegetables most days I would feel…”*		
…I stand up for my beliefs.	.634	.437
…I am a role model for other parents.	.507	.560
…I have self-respect.	.642	.509
…I am making a contribution.	.512	.563
…I am being honest and fair.	.580	.501

**Legend**: Response Scale: 1 = Disagree, 2 = Neither Agree nor Disagree, 3 = Agree; for the Pearson correlations between subscales: *** = p < 0.001.

Using the same three category agreement response (1 = Disagree, 2 = Neither Agree nor Disagree, 3 = Agree), three autonomy items, twenty-six barrier items, and four desire (similar to the intrinsic motivation construct) items were generated. (See individual items in Tables 9, 10, and 11.)

**Table 9:** Items and factor loadings from an exploratory single factor solution of autonomy toward use of vegetable parenting practices and confirmatory factory analysis model fit criteria

**Autonomy items**	**Factor 1 loading**
	**Autonomy**
It is my choice to encourage my child to eat at least 3 portions of vegetables most days.	**.777**
I have a choice about what vegetables to offer my child.	**.701**
I feel like I have to get my child to eat at least 3 portions of vegetables most days.	**.500**
Eigenvalue	1.346
Variance explained	44.9%
*Model Fit Indices from a Single Factor Confirmatory Factor Analysis*	
*X*^*2*^	Not positive definite
df	
*p*	
RMSEA	
SRMR	
CFI	
TLI	

**Table 10:** Items and factor loadings from an exploratory three factor solution of perceived barriers toward use of vegetable parenting practices and confirmatory factory analysis model fit criteria

**Perceived Barrier Items**	**Factor 1 loadings**	**Factor 2 loadings**	**Factor 3 loadings**
	**Child doesn’t like vegetables**	**Respondent doesn’t like vegetables**	**Cost of vegetables**
***Child doesn’t like vegetables***			
Getting my child to eat vegetables at meals is difficult.	**.846**	.189	.043
My child doesn’t like the taste of vegetables.	**.786**	.131	.073
My child does not like the texture of vegetables.	**.781**	.180	.029
My child prefers other foods over vegetables.	**.732**	-.001	.117
My child is a picky eater.	**.697**	.042	.153
My child doesn’t eat vegetables as snacks.	**.649**	.088	.300
It is hard to find vegetables my child likes in stores.	**.646**	.169	.035
It is hard to find vegetables my child likes at restaurants or fast food places.	**.614**	.144	.165
***Respondent doesn’t like vegetables***			
I don’t like vegetables myself.	.027	**.804**	.108
No one in my family eats vegetables.	.038	**.801**	.158
I don’t like the taste of vegetables.	.049	**.796**	.038
I don’t know how to cook vegetables.	.251	**.651**	.175
I don’t like to cook vegetables.	.147	**.645**	.362
It is difficult to find recipes for vegetables.	.345	**.580**	.060
I don’t usually have vegetables at home.	.236	**.559**	.250
I usually forget to serve vegetables to my child.	.212	**.533**	.367
It is not important that my child eats vegetables.	-.024	**.502**	.032
***Cost of vegetables***			
Fresh vegetables spoil too fast.	.050	.028	**.716**
I only have a small amount to spend on vegetables.	-.029	.268	**.653**
Vegetables are expensive.	.123	.126	**.589**
I usually don’t buy fresh vegetables.	.152	.341	**.502**
It takes too long to make a vegetable snack when my child is hungry.	.356	.163	**.482**
Eigenvalue	8.378	3.107	1.560
Variance explained	32.2%	11.9%	6.0%
	Pearson correlation		
***Child doesn’t like vegetables***		0.391***	0.382***
***Respondent doesn’t like vegetables***			0.506***
***Cost of vegetables***			
*Model fit indices from a three factor confirmatory factor analysis*			
*X*^*2*^	390.106		
df	206		
*p*	<0.001		
RMSEA	0.054		
SRMR	0.09		
CFI	0.964		
TLI	0.96		
***Items not included in a final solution***			
Vegetables do not fill my child up.	.237	.255	.278
I usually don’t serve vegetables for snacks.	.486	.057	.482
I don’t have time to prepare vegetables.	.190	.537	.475
I don’t know how to prepare vegetables so that everyone in the family will eat them.	.406	.504	.298

**Legend**: Response Scale: 1 = Disagree, 2 = Neither Agree nor Disagree, 3 = Agree; for the Pearson correlations between subscales: *** = p < 0.001.

**Table 11:** Items and factor loadings from an exploratory single factor solution of desire toward use of vegetable parenting practices and confirmatory factory analysis model fit criteria

**Desire items**	**Factor 1 loading**
***Desire:****“Encouraging my child to eat vegetables is…”*	
…hard.	**.860**
…frustrating.	**.847**
…enjoyable.	**-.776**
…rewarding.	**-.591**
Eigenvalue	2.408
Variance explained	60.2%
*Model fit indices from a single factor confirmatory factor analysis*	
*X*^*2*^	3.217
df	1
*p*	0.073
RMSEA	0.085
SRMR	0.015
CFI	0.999
TLI	0.995

Twenty-one intention items were generated starting with the stem “In the next month I plan to…”, using a three category intention response (1 = Will Not Do, 2 = May or may Not Do, 3 = Will Do). (See individual items in Table 12.)

**Table 12:** Items and factor loadings from an exploratory four factor solution of intentions toward use of vegetable parenting practices and confirmatory factory analysis model fit criteria

**Intentions items**	**Factor 1 loadings**	**Factor 2 loadings**	**Factor 3 loadings**	**Factor 4 loadings**
	**Authoritative parenting intentions**	**Active child involvement intentions**	**Controlling parenting intentions**	**Permissive parenting intentions**
***Authoritative parenting intentions:****“In the next month I plan to…”*				
…encourage my child to try a couple of bites of a vegetable.	**.846**	.150	-.056	.002
…tell my child eating vegetables will make them strong and healthy.	**.805**	.134	.059	-.095
…tell my child that vegetables taste good.	**.769**	.058	.058	-.017
…praise my child when I see them eat vegetables.	**.688**	.107	.002	-.009
…set an example by eating vegetables myself.	**.650**	.088	-.056	-.070
…give my child vegetables they like.	**.628**	.302	-.096	.024
***Active child involvement intentions:****“In the next month I plan to…”*				
…ask my child to help with vegetable preparation.	.043	**.822**	-.036	-.007
…ask my child to choose the vegetables for meals and snacks.	.090	**.810**	-.009	.065
…ask my child to help select vegetables at the grocery store.	.204	**.785**	-.042	.142
…allow my child to serve themselves vegetables.	.122	**.684**	-.111	.291
…make eating vegetables fun, like cutting into shapes.	.116	**.675**	.214	-.036
…buy vegetables for snacks instead of cookies, chips and candy.	.319	**.604**	.054	-.105
***Controlling parenting intentions:****“In the next month I plan to…”*				
…keep my child from going to play if they don’t eat their vegetables.	-.009	-.112	**.813**	.009
…insist my child sit at the table until they eat their vegetables.	.024	.041	**.747**	-.044
…tell my child how much effort it took to make the vegetables	-.035	.094	**.720**	.111
…beg my child to eat their vegetables.	-.122	-.103	**.569**	.192
…tell my child that their favorite cartoon characters eat vegetables.	.205	.196	**.452**	.099
***Permissive parenting intentions:****“In the next month I plan to…”*				
…let my child eat when they want to eat.	-.027	.039	.018	**.809**
…make something different if my child does not like what was served.	-.040	.093	.162	**.779**
Eigenvalue	5.06	3.08	2.24	1.40
Variance explained	24.1%	14.6%	10.7%	6.7%
	Pearson correlation			
***Authoritative parenting intentions***		0.367***	0.007	−0.071
***Active child involvement intentions***			0.066	0.141*
***Controlling parenting intentions***				0.202***
***Permissive parenting intentions***				
*Model fit indices from a four factor confirmatory factor analysis*				
*X*^*2*^	342.938			
df	140			
*p*	<0.001			
RMSEA	0.069			
SRMR	0.108			
CFI	0.979			
TLI	0.974			
***Items not included in a final solution:****“In the next month I plan to…”*				
…schedule meals for my child.	.379	.399	.128	-.138
…offer my child something to eat to stop a temper tantrum.	-.184	.039	.480	.611

**Legend**: Response Scale: 1 = Disagree, 2 = Neither Agree nor Disagree, 3 = Agree; for the Pearson correlations between subscales: * = p < 0.05, *** = p < 0.001.

### Other measures

In a separate manuscript [8] with data from this internet survey, we reported confirmatory factor analyses on only the EVPP and IVPP (separate items developed in the same way) with the same sample indicating the most interpretable structure had separate (completely independent) two-level factor structures [8]. For the analyses reported herein, the values for the 14 effective items were summed (EVPP sum (possible range: 14–102), Cronbach’s alpha = 0.69) and the 14 ineffective items were summed (IVPP sum (possible range: 14–102), Cronbach’s alpha = 0.60) to obtain unweighted composite scales. Participants reported gender of participating parent, gender of selected child, ethnicity of parent, highest household educational attainment, and annual household income using standard questions.

### Analyses

The items for each of the 11 scales were submitted to exploratory factor analysis (principal components) with a varimax rotation, using the scree plot criterion for factor extraction using SPSS [24]. Exploratory factor analysis was used for data reduction and to examine whether the 11 scales were uni-dimensional or consisted of several underlying factors (i.e. subscales). Items not loading on a factor (factor loading <0.4) or loading on more than one factor were deleted from the scale and the analysis reconducted with the reduced set of items. Percentage of variance in the items accounted for by a factor was estimated using the eigenvalues. The exploratory factor structure was submitted to a confirmatory factor analysis (structural equation modeling) using the same sample to obtain model fit indices using Mplus [25]. Hu and Bentler’s two-index presentation strategy [26] were employed to access the data-model fit. The combinational rules include 1) TLI of 0.96 or higher and an SRMR of 0.09 or lower; 2) RMSEA of 0.06 or lower and an SRMR of 0.09 or lower 3) CFI of 0.96 or higher and an SRMR of 0.09 or lower. Subscale means and standard deviations were calculated and range of scores noted. Cronbach’s alpha and the average inter-item correlation [27] were calculated for each subscale. When the number of items is small (e.g. 5 or less), an average inter-item correlation between 0.15 and 0.50 is considered an indication of acceptable internal consistency depending on the generality-specificity of the construct [27]. Pearson correlations were calculated among MGDVPP subscales and between each MGDVPP subscale and composite scales of EVPP and IVPP.

## Results

406 participants provided informed consent, entered the questionnaire website and initiated the questionnaire; 16 participants were deleted because they did not have a 3 to 5 year old child, or the child did not spend most days with that parent or guardian. Complete data were obtained from 307 participants. Since the demographic questions were at the end of the survey, we do not have the necessary data to compare the 83 participants who provided incomplete data with the 307 who provided complete data. Almost 90% of respondents were female, but slightly more of the children were male (53.1%) (Table 13). A plurality of respondents were white (37.1%), with representation from all major ethnic groups in Houston (19.5% Black/African American, 10.1% Hispanic, 14.0% Asian, and 19.2% Other). The sample was well educated with over half (64.5%) having a college degree or more. Over half (54.1%) had an annual household income of $60,000 or higher. The mean (±sd) Effective Vegetable Parenting Scale score was 23(±3.6); and the mean (±sd) Ineffective Vegetable Parenting Scale score was 34.4 (±3.1) [8]. Eleven scales with 192 items were submitted to exploratory and confirmatory factor analyses with 164 items retained in 29 subscales. The psychometric results for the eleven scales are found in Tables 1, 14, and 3 through 12.

**Table 13:** Sample demographic characteristics

	**n**	**%**
**Total**	307	100.0
**Gender of parent**		
Male	33	10.7
Female	274	89.3
**Gender of child**		
Male	163	53.1
Female	144	46.9
**Ethnicity of parent**		
Black/African American	60	19.5
White	114	37.1
Hispanic	31	10.1
Asian	43	14.0
Other	59	19.2
**Household highest educational attainment**		
HS grad or less	30	9.7
Technical school	11	3.6
Some college	67	21.8
College graduate	96	31.3
Postgrad study	102	33.2
Missing	1	0.3
**Annual household income (2009)**		
< $10 K	11	3.6
$10 K - $19 K	16	5.2
$20 K – $39 K	56	18.2
$40 K - $59 K	58	18.9
≥ $60 K	166	54.1

**Table 14:** Means, standard deviations, ranges, number of items, Cronbach’s alphas and correlations for subscales from a Model of Goal Directed Vegetable Parenting Practices (MGDVPP)

**MGDVPP scales**	**MGDVPP subscales**	**Means**	**SD**	**Ranges**	**Number of Items**	**Cronbach’s alphas**	**Average interitem correlation**	**Pearson correlations**	
								**Effective vegetable parenting practices**	**Ineffective Vegetable parenting practices**
Attitudes	Health benefits of vegetables	16.14	2.03	9 - 18	6	0.72	0.31	−0.08	−0.14*
	Negative effects of vegetables	7.42	1.73	6 - 15	6	0.66	0.25	0.08	−0.16**
	Benefits of vegetables other than Health	11.58	0.94	7 - 12	4	0.66	0.36	−0.07	−0.02
Norms	Descriptive norms	3.86	0.83	2-6	2	0.13	0.07	−0.10	−0.15**
	Normative expectations	11.86	5.17	1-18	2	0.71	0.55	−0.08	−0.29***
Perceived	Control of positive influences on vegetable consumption	34.46	4.37	17 - 39	13	0.85	0.32	−0.37***	0.002
Behavioral	Control of negative influences on vegetable consumption	16.93	4.29	11 - 32	11	0.82	0.31	0.05	−0.26***
Control	Control of negative parenting practices	7.55	1.80	4 - 12	4	0.54	0.22	−0.06	−0.45***
Anticipated Emotions	Positive parent emotional response to child vegetable refusal	9.69	2.84	8 - 23	8	0.92	0.58	−0.08	0.04
	Negative parent emotional response to child vegetable acceptance	4.82	1.50	4 - 11	4	0.83	0.62	0.02	−0.04
	Negative parent emotional response to child vegetable refusal	17.90	3.87	8 - 24	8	0.79	0.32	0.13*	−0.35***
	Positive parent emotional response to child vegetable acceptance	11.38	1.17	4 - 12	4	0.66	0.41	−0.05	−0.2***
Habit	Habit of active child involvement in vegetable selection	10.98	3.04	6 - 18	6	0.83	0.45	0.6***	−0.1
	Habit of controlling vegetable practices	11.80	2.13	5 - 15	5	0.68	0.31	0.11	0.51***
	Habit of positive vegetable environment	3.59	0.95	3 - 8	3	0.67	0.43	0.44***	−0.12*
	Habit of positive vegetable communications	6.92	1.74	5 - 13	5	0.60	0.27	0.44***	0.08
Competence/Self Efficacy	Advanced vegetable parenting self efficacy	19.27	3.87	8 - 24	8	0.85	0.41	−0.38***	0.08
	Preliminary vegetable parenting self efficacy	27.99	2.50	19 - 30	10	0.76	0.27	−0.28***	0.1
Relatedness	Parent values	7.72	2.16	4 - 12	4	0.81	0.52	−0.13*	−0.21***
	Child wellness	8.26	1.15	3 - 9	3	0.61	0.36	−0.08	−0.11
Autonomy	Choice	7.92	1.06	4 - 9	3	0.31	0.17	−0.23***	−0.05
Perceived Barriers	Child doesn’t like vegetables	14.69	4.88	8 - 24	8	0.88	0.49	−0.35***	0.2***
	Respondent doesn’t iike vegetables	11.14	3.30	9 - 26	9	0.85	0.42	0.39***	−0.24***
	Cost of vegetables	7.53	2.34	5 - 15	5	0.67	0.30	0.32***	−0.22***
Desire	Desire	9.01	2.27	4 - 12	4	0.78	0.46	0.23***	−0.23***
Intentions	Authoritative parenting intentions	17.50	1.31	11 - 18	6	0.83	0.47	−0.14*	0.03
	Active child involvement intentions	16.05	2.41	6 - 18	6	0.84	0.48	−0.33***	0.12*
	Controlling parenting Intentions	9.54	2.59	5 - 15	5	0.71	0.33	−0.01	−0.49***
	Permissive parenting intentions	3.66	1.28	2 - 6	2	0.61	0.44	0.01	−0.18**

**Legend:** * < .05, ** < .01, *** < .001; Response Scale: 1 = Disagree, 2 = Neither Agree nor Disagree, 3 = Agree.

Acceptable fit was obtained for most of the scales, and predictive validity with EVPP and/or IVPP was obtained for 25 of 29 subscales (Table 14). Exceptions to acceptable fit include the below. Confirmatory factor analysis revealed marginally acceptable fit for the four factor structure among Anticipated Emotions items (bottom of Table 5). Cronbach’s alphas varied from 0.66 to 0.92 and average inter-item correlations ranged from 0.32 to 0.62 (Table 14) suggesting the internal consistency for the subscales with 4 items were acceptable. Confirmatory factor analysis revealed marginally acceptable model fit for the two factor structure among Competence/Self Efficacy items (bottom of Table 7). Cronbach’s alphas for the two subscales, however, were 0.85 and 0.76. The confirmatory factor analysis for the three Autonomy items could not attain positive definite status (Table 9). Cronbach’s alpha for the scale was 0.31 while the average interitem correlation was 0.17 which was at the lower end of the range of acceptable (Table 14). Despite this low internal consistency reliability, it was significantly inversely correlated with EVPP (r = −0.23, p < 0.001) (Table 14). Confirmatory factor analysis revealed marginally acceptable fit for the four factor solution among Intentions items (bottom of Table 12).

## Discussion

Exploratory factor analyses of each of the 11 original scales separately indicated there were 29 subscales with 2 to 13 items per subscale; three subscales had 10 or more items; 12 subscales had 4 items or less. Model fit was acceptable in most cases. Cronbach’s alphas for the subscales ranged from 0.13 to 0.92 with 17 being 0.70 or higher. Most alphas <0.70 included only three or four items, but acceptable average inter-item correlations [27]. Twenty-five of 29 subscales significantly bivariately correlated with composite effective or ineffective VPP.

To our knowledge, this is the first report of the psychometric characteristics of theory based scales and subscales to predict a parent’s use of VPP. Most studies using TPB [28] or MGDB [12-15] used single dimensional scales for each predictive construct. Our approach, alternatively, found single dimensions did not adequately fit the items for most scales/constructs. Using the scree plot criterion and interpretability, exploratory analyses obtained one to four dimensions per scale/construct.

A number of subscales (12/29) had internal reliabilities less than 0.7 which is generally considered low [29]. Low scale reliability attenuates relationships with other variables [29]. Most of these subscales included only 3 or 4 items. Since Cronbach’s alpha is sensitive to the number of items, for subscales with few items an average inter-item correlation in the range of 0.15 to 0.50 is considered an indicator of an acceptable level of internal consistency [27]. Of the 12 subscales with 4 items or less, the average inter-item correlation was in the acceptable range for 9 of them, and for 2 it exceeded the range. This suggests that a true dimension was detected, but additional work is needed to generate new items to expand the subscale, test dimensionality, and re-assess the psychometrics of the new subscales and scales. Since norms have a long history as a part of the Theory of Planned Behavior [28], the Descriptive Norms subscale should be retained, but further developed to enhance its reliability.

Factorial validity (CFA) could not be established for four scales even though internal consistency reliability was acceptable for all but the Autonomy scale. The CFA for the Autonomy items could not achieve positive definite status. Several direct estimation methods (weighted least squares, mean-adjusted weighted least squares, and variance-adjusted weighted least squares) were tried, but to no avail. The low Cronbach’s alpha (0.31), the consistently low corrected item total correlations (0.15, 0.19, 0.25), and the low average inter-item correlation (0.17) suggested that autonomy is a complex construct and the items we included tapped multiple dimensions, which were not highly interrelated. Since Autonomy included only three items, more development of this scale and possible subscales is warranted.

We had no theoretical foundation for theoretically deducing which MGDVPP subscales would correlate with EVPP or IVPP. Despite some low reliabilities, 25 of 29 subscales correlated with one or the other of the composite EVPP or IVPP. Parent Values (a Relatedness subscale) significantly inversely correlated with EVPP and IVPP. Similarly, most Intentions subscales inversely correlated with EVPP and IVPP. It is likely that respondents did not know which VPP were effective or ineffective, which may have influenced these relationships. It is possible that respondents thought the Intention items should only be answered positively if they were not already doing it, but intending to do it in the next month. Future research with these scales will need to address these issues.

Thirty intercorrelations among subscales were tested; 9 were not significant; 5 were significant at p < 0.05, 1 at p < 0.01, and 15 at p < 0.001. The subscales tended to be intercorrelated in expected directions within scales. The highest correlation was 0.51 between the Perceived Barriers of Respondent Doesn’t Like Vegetables and Cost of Vegetables. Intersubscale correlations will need to be validated in future studies. While not high enough in this sample to constitute multicollinearity, it is possible that future studies will identify different dimensions combining subscales in the current sample.

The strengths of this research include use of a broad innovative theoretical model to predict behaviors (here vegetable parenting practices); qualitative methods to generate items from the target group; and narrowly focused on parents of a developmentally similar age group. A number of limitations exist. The sample was limited in size and diversity. Further research is needed with larger samples to permit more sophisticated analyses and with more diverse samples to test generalizability across gender, ethnicity, and socioeconomic status. The internet survey method did not allow collecting and matching data from a second time point, thereby precluding an assessment of test-retest reliability; and the same sample was employed for exploratory and confirmatory factor analyses. Predictive validity was tested with cross-sectional data; these need to be verified with longitudinal data. Additional research with larger samples should use Item Response Modeling (IRM) to better understand the sequencing of items, difficulties across the latent constructs, the matching of item distributions with participant distributions, and to assess differences in item responses (i.e. differential item functioning) by demographic characteristics [30,31]. IRM would also permit efficient reduction of items in the subscales with larger numbers by identifying items redundant at location along the latent variable [17]. Twenty-nine subscales were identified. While model testing research should include all 29 to verify (or disconfirm) the current findings, investigators with a more practical or applied intent may wish to select subscales most clearly related to their efforts. The four subscales that did not correlate with EVPP or IVPP, and the ones that correlated in unexpected directions, need further testing in other samples.

Although further development is warranted, these scales and subscales can be used in studies attempting to understand why parents might use effective and ineffective vegetable parenting practices.

## Abbreviations

BCM: Baylor College of Medicine; CFI: Comparative fit index; CNRC: Children’s Nutrition Research Center; EVPP: Effective vegetable parenting practices; IRM: Item response modeling; IVPP: Ineffective vegetable parenting practices; MGDB: Model of goal directed behavior; MGDVPP: Model of goal directed vegetable parenting practices; RMSEA: Root mean square error of approximation; SRMR: Standardized root mean-square residual; TLI: Tucker lewis index; VPP: Vegetable parenting practices.

## Competing interests

The authors declare that they have no competing interests.

## Authors’ contributions

TB was principal investigator for the overall project and wrote the first draft of the manuscript. AB was the project manager. T-AC was the data manager and statistician. DT, TO, and SH contributed conceptually to the measures. CD participated in review of analyses. JB was the project coordinator. All authors reviewed, critiqued and approved this manuscript.
